# Predictors of Survival in Esophageal Squamous Cell Carcinoma with Pathologic Major Response after Neoadjuvant Chemoradiation Therapy and Surgery: The Impact of Chemotherapy Protocols

**DOI:** 10.1155/2016/6423297

**Published:** 2016-10-04

**Authors:** Chia-Ying Li, Pei-Ming Huang, Pei-Yi Chu, Po-Ming Chen, Mong-Wei Lin, Shuenn-Wen Kuo, Jang-Ming Lee

**Affiliations:** ^1^Department of Surgery, Show Chwan Memorial Hospital, Changhua City, Taiwan; ^2^Division of Thoracic Surgery, Department of Surgery, National Taiwan University Hospital and National Taiwan University College of Medicine, Taipei, Taiwan; ^3^Department of Pathology, Show Chwan Memorial Hospital, Changhua, Taiwan; ^4^School of Medicine, College of Medicine, Fu-Jen Catholic University, New Taipei City, Taiwan; ^5^National Institute of Cancer Research, National Health Research Institutes, Tainan, Taiwan; ^6^Research Assistant Center, Show Chwan Memorial Hospital, Changhua, Taiwan; ^7^Institute of Molecular and Genomic Medicine, National Health Research Institutes, Miaoli, Taiwan

## Abstract

Tumor recurrence is an important problem threatening esophageal cancer patients after surgery, even when they achieve a pathologic major response (pMR) after neoadjuvant concurrent chemoradiation therapy (CCRT). The predictors related to overall survival and disease progression for these patients remain elusive. We aimed to identify factors that predict disease progression and overall survival in esophageal squamous cell carcinoma (SCC) patients who achieve a pMR after neoadjuvant CCRT followed by surgery. We conducted a retrospective study to analyze the factors influencing survival and disease progression after esophagectomy for esophageal cancer patients who had a major response to CCRT, which is defined by complete pathological response or microscopic residual disease without lymph node metastasis. From our study cohort, 285 patients underwent CCRT and subsequent esophagectomy; 171 (60%) of these patients achieved pMR. After excluding patients with lymph node metastases, incomplete clinical data, and adenocarcinomas, we enrolled 117 patients in this study. We found that the CCRT regimen was the only factor that influenced overall survival. The overall survival of the patients receiving taxane-incorporated CCRT was superior to that of patients receiving traditional cisplatin and 5-fluorouracil (PF) (*P* = 0.011). The CCRT regimen can significantly influence the clinical outcome of esophageal SCC patients who achieve pMR after neoadjuvant CCRT and esophagectomy. Incorporation of taxanes into cisplatin-based CCRT may be associated with prolonged survival.

## 1. Introduction

Esophageal cancer is the fifth leading cause of cancer deaths worldwide [1]. Unfortunately, most esophageal cancer patients present with advanced disease at diagnosis, and the outcome is poor (5-year survival rate: 10–22%) when surgery alone is used to treat such patients [2–5]. Several studies have shown that neoadjuvant concurrent chemoradiation therapy (CCRT) followed by surgery affords better locoregional control, disease-free survival, and overall survival than surgery alone [3, 6, 7]. The rationale for neoadjuvant CCRT includes eradication of micrometastasis and improvement of primary tumor resectability [1].

Squamous cell carcinoma (SCC) is more common than adenocarcinoma in the Far East [1]. These tumors differ not only histologically, but also in the pathogenesis, tumor location, and clinical outcome [8].

The pathological response to CCRT is associated with disease recurrence and overall survival, and 60–70% of patients achieve a pathologic major response (pMR) after CCRT [1, 4]. Although patients with pMR are reported to have a better prognosis after surgery than those without, approximately 40% of the former develop local or systemic recurrence and die from disease progression after treatment [1].

Only a few studies have assessed the predictors of survival or recurrence in such patients. It was found that a pretreatment disease stage of T3-T4 was a poor prognostic factor [9]. Nonetheless, CCRT was administered to these patients in most cases. The prognostic factors for patients with major downstaging of the tumor after CCRT are unknown. We, therefore, addressed this issue in the present study.

## 2. Materials and Methods

### 2.1. Patients

This study was based on a retrospective chart review of patients who underwent neoadjuvant CCRT for esophageal cancer and esophagectomy, between January 1997 and January 2010 at the National Taiwan University Hospital, Taipei, Taiwan. The pre-CCRT staging work-up included upper gastrointestinal series, computed tomography (brain, chest, and abdomen), and panendoscopy. Endoscopic ultrasonography and positron emission tomography were also performed in some patients. After staging work-up, a multidisciplinary team evaluated each patient to assess the resectability of the tumors, and then the patients underwent neoadjuvant CCRT. The post-CCRT staging reevaluation strategy was identical to the initial staging work-up. If surgery was not recommended due to underlying medical problems or advanced disease, patients underwent definitive CCRT. This study was approved by the ethics committee of the Institutional Review Board (IRB) in Show Chwan Hospital. Informed consents were obtained from all sample donors in accordance with the Declaration of Helsinki.

### 2.2. Neoadjuvant CCRT

For neoadjuvant CCRT, radiation was delivered using the standard anteroposterior/posteroanterior field technique. The radiation field included the supraclavicular region if the lesion was above the carina and included the celiac trunk if the lesion was near the esophageal-gastric junction. The margin from the gross tumor to the field borders was 2 cm circumferentially and 5 cm superiorly/inferiorly. The radiation dose applied to the initial irradiated volume was 40 Gy. Radiation (2 Gy per fraction, once daily) was delivered to the isocenter in 5 fractions per week [3]. The chemotherapy regimens of cisplatin and 5-fluorouracil (PF) or taxanes and cisplatin (TP) were administered concurrently with radiotherapy. The chemotherapy regimen was chosen by the physician at the time of patient evaluation. The PF regimen consisted of low-dose cisplatin (6 mg/m^2^ with 30–60 min intravenous infusion, on days 1–5 of weeks 1–4) and continuous 24 h intravenous infusion of 5-FU (225 mg/m^2^ on days 1–7 of weeks 1–4) [3, 10]. The TP regimen consisted of twice-weekly administration of paclitaxel (35 mg/m^2^ with 1 h intravenous infusion, on days 1 and 4 of weeks 1–4) and cisplatin (15 mg/m^2^ with 1 h intravenous infusion, on days 2 and 5 of weeks 1–4) [3, 11].

Some patients initially received induction chemotherapy, which included modified TPFL (Taxotere, cisplatin, 5-FU, and leucovorin) or TP-HDFL (Taxotere, cisplatin, high-dose 5-FU, and leucovorin). The modified TPFL regimen comprised docetaxel (40 mg/m^2^ with 1 h intravenous infusion, on day 1), cisplatin (35 mg/m^2^ with 3 h intravenous infusion, on day 1), 5-FU (2200 mg/m^2^ with 46 h intravenous infusion, on day 1), and leucovorin (300 mg/m^2^ with 46 h intravenous infusion, on day 1). The TP-HDFL regimen consisted of paclitaxel (80 mg/m^2^ with 1 h intravenous infusion, on days 2 and 8), cisplatin (35 mg/m^2^ with 24 h intravenous infusion, on days 2 and 9), 5-FU (2000 mg/m^2^ with 24 h intravenous infusion, on days 2 and 9), and leucovorin (300 mg/m^2^ with 24 h intravenous infusion, on days 2 and 9). One cycle of induction therapy is approximately 2-3 weeks. The patients received 1–3 cycles of induction therapy and then the standard neoadjuvant CCRT (TP regimen). These treatment schedules of induction chemotherapy followed by neoadjuvant CCRT (TP regimen) and the standard neoadjuvant CCRT with TP regimen were defined as taxane-incorporated CCRT.

During CCRT, the dosage of chemotherapy was modified according to the patients' general condition and results of laboratory tests. In most cases, surgical interventions were performed 4–8 weeks after the completion of CCRT.

### 2.3. Surgery and Pathology

The options for surgical resection, including subtotal esophagectomy and regional lymph node dissection, were performed via right thoracotomy, right video-assisted thoracoscopic surgery (VATS), or left thoracoabdominal incision, depending on the tumor location and the surgeon's discretion. Esophageal reconstruction was preferably performed using a gastric tube; if the gastric tissue was unusable, a colon flap was used.

The response to CCRT was evaluated according to the findings of pathological examination of the surgical specimens. A pathologic complete response (pCR) was defined as the absence of cancer cells in the specimens upon microscopic examination. Microscopic residual disease (MRD) implied that no tumor lesion was detected on macroscopic observation, but microscopic examination revealed some residual tumor cells, where the residual tumor cells are distributed across an area that is <10% of the area with CCRT-related tissue injury. Patients achieving a pCR or MRD after CCRT were defined as having a pMR. The presence of lymph node metastasis was excluded in our analysis. The staging was performed according to the criteria defined by the 2002 American Joint Committee on Cancer Staging system (AJCC).

### 2.4. Data Analysis

Comparison of categorical variables was drawn using the chi-square test or Fisher's exact test. The intervals of survival or disease progression were defined from the date of surgery to the date of death, last follow-up, or disease progression. Kaplan-Meier calculations were used to compare progression-free survival and overall survival. The association of factors with progression-free survival and overall survival was analyzed using Cox proportional hazards model including the possible factors. Differences were considered statistically significant when the two-sided *P* value was less than 0.05. All analyses were performed using the Statistical Package for Social Sciences (SPSS) version 18.0 (SPSS Institute, Chicago, IL).

## 3. Results

### 3.1. General Characteristics of the Study Participants

Treatment comprising neoadjuvant CCRT followed by esophagectomy was administered to 285 patients with locally advanced esophageal cancer. Of these patients, 171 (60%) achieved pMR. After excluding patients with lymph node metastases and unavailable CCRT regimens, 119 patients remained. Among these patients, 117 had SCC and 2 had adenocarcinomas, the latter of which were excluded from our study.

The mean age of the 117 patients (107 men, 10 women) was 58 y (range, 37–77 y). Of the 117 patients, 101 had stage T3-T4 tumors and 16 patients had stage T2 tumors. PF was administered to 50 patients, and taxane-incorporated CCRT was administered to 67 patients. After neoadjuvant CCRT followed by esophagectomy, the pathologic stage was T3 in 10 patients and T0–T2 in 107 patients.

The median postoperative follow-up period was 21.54 months. Postoperative mortality was defined as patient death prior to hospital discharge, and it occurred in 12 cases (10.3%). Local or systematic cancer recurred in 61 patients during the follow-up period. Postoperative mortality and cancer recurrence were considered as disease progression. Postoperative complications included anastomosis leakage, wound infection, and pulmonary conditions. In all cases of postoperative mortality, death occurred due to complication-related sepsis and multiple organ failure.

Neoadjuvant CCRT complications, which occurred in 34.2% of the patients receiving this therapy, included hematologic suppression, infection, esophagitis, and other CCRT-related discomforts.

The clinical characteristics of patients with or without disease progression, listed in Table 1, showed that there were no significant differences between these 2 patient groups, except for the chemotherapeutic regimen; patients receiving PF had a higher rate of disease progression than those receiving taxane-incorporated CCRT (*P* = 0.002). We stratified the 117 patients according to the chemotherapy regimens, as summarized in Table 2. There was a statistically significant difference in the age and rate of disease progression between the 2 groups (*P* = 0.001 and 0.002, resp.); patients in the taxane-incorporated CCRT group tended to be younger and had a lower disease progression rate. We believe that the age difference might be related to the initial patient selection.

### 3.2. Survival Data

Of a total of 117 esophageal cancer patients, 72 patients underwent disease progression after CCRT followed by surgery. Among 72 patients, 24 patients were diagnosed as local recurrence and 48 patients diagnosed as systemic recurrence (metastasis) (see Supplementary Table  1 in Supplementary Material available online at http://dx.doi.org/10.1155/2016/6423297). Among the clinic-pathological parameters studied, including age, gender, T staging, CCRT response, tumor site, op. complication, and CCRT regimen, the parameters were not correlated with local recurrence and systemic recurrence (metastasis) (Supplementary Table 1).

**Figure 1:**
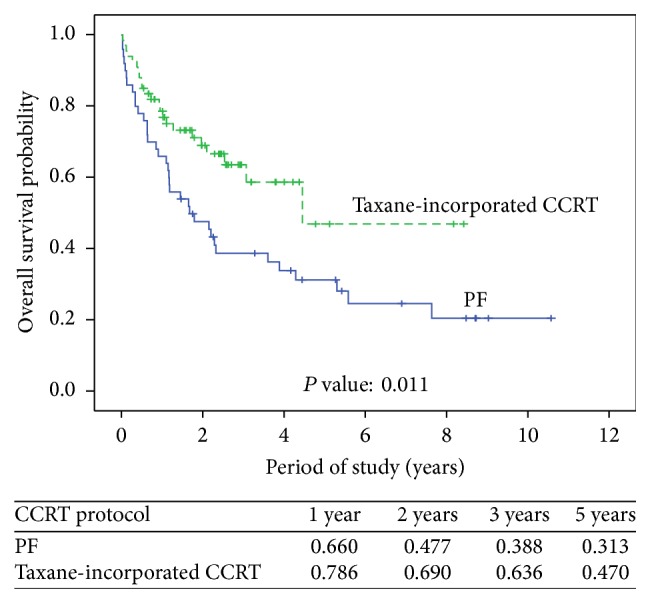
Overall survival based on neoadjuvant chemotherapeutic regimens (*P* = 0.011).

**Figure 2:**
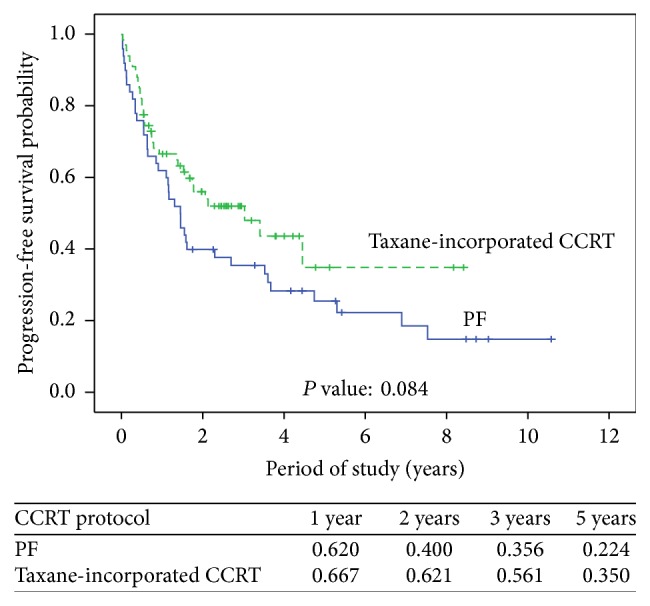
Progression-free survival based on neoadjuvant chemotherapeutic regimens (*P* = 0.084).

We analyzed the association of factors with overall survival and progression-free survival (Table 3). Significant impacts of postoperative complications (*P* = 0.014) and different CCRT regimen (*P* = 0.013) were noted for overall survival. Even after adjusting for other factors, postoperative complications and CCRT regimen remained associated with overall survival (*P* = 0.024 and 0.017, resp.). Progression-free survival was significantly associated with postoperative complications (*P* = 0.034). However, the significance was borderline after adjusting for other factors (*P* = 0.066). Kaplan-Meier estimates of overall survival and progression-free survival in the 2 groups with different CCRT regimens are shown in Figures 1 and 2, respectively. The mean overall survival and progression-free survival in the PF group were 1.68 and 1.45 years, respectively. The mean overall survival and progression-free survival in the taxane-incorporated CCRT group were 4.40 and 3.04 years, respectively. Patients who received taxane-incorporated CCRT had better overall survival than those who received PF (Figure 1, *P* = 0.011). There were no significant differences in the progression-free survival of patients in the PF group and taxane-incorporated CCRT group (Figure 2, *P* = 0.084).

## 4. Discussion

Neoadjuvant CCRT followed by surgical resection has been proven to yield better results than surgical resection alone, and it has become the standard therapy for locally advanced esophageal cancer, although other studies dispute this [2–7, 12–14]. Many studies have assessed the predictors of cancer recurrence and survival after neoadjuvant CCRT and surgical resection. Tumor response to CCRT has been identified as a significant predictive factor, which suggests that it should be used in addition to the current pTNM staging system for a more precise prediction of long-term survival [1, 2, 5, 11, 15–17].

After CCRT, 60–70% of patients achieve a pMR, including pCR and MRD. pCR is defined as the absence of evidence for residual and viable tumor cells upon microscopic examination of both the resected esophageal specimen and lymph nodes after neoadjuvant CCRT [1, 4]. Patients achieving pCR had longer overall survival and fewer disease recurrences than those who achieved a partial or no response [2]. Only 15–36% of patients achieved a pCR after neoadjuvant CCRT. However, 22–30% of the patients still suffered recurrences, and survival time after recurrence is short regardless of the pathologic response [2, 18]. The pCR rate observed in this study (23.7%) is comparable to that reported in previous studies.

MRD is characterized by occasional residual cancer cells that appear scattered in resected esophageal specimens examined microscopically. Patients with residual tumor cells in lymph node tissue were excluded from our study because node-positive status is an indicator of poor prognosis [19]. Small foci of residual tumor cells might be easily missed during histopathological examination, particularly when en bloc tumor embedding is not performed and only a few sections are evaluated. This could result in the underestimation of MRD and the overestimation of a pCR. Therefore, in our study, no statistically significant difference was noted in the outcomes between patients achieving pCR and those achieving MRD (Tables 1 and 3), which is consistent with the findings of previous studies [19–21]. In our study, the proportion of local recurrences in the patients with pMR was 30.3%, which is a little higher than that reported previously (19–28%) [2, 22]. This might be attributed to the high proportion of patients with locally advanced cancer included in our study (94.4% of patients had pre-CCRT tumor stages of T3-T4). In our study, only 2 of the 119 tumors were adenocarcinomas, which may be related to the epidemiological features of this cancer in the Far East [11]. Another reason may be that SCC is considered a predictor of pMR and is associated with better survival than non-SCC [3, 5].

According to the study conducted by Chao and colleagues, tumor stage of T3-T4 before the initiation of CCRT is an adverse risk factor for tumor recurrence in esophageal SCC patients who achieve pCR [9]. We found that the CCRT regimen was another factor that could influence the overall survival.

Initially, taxanes were used in the palliative therapy for patients with ovarian and breast cancers resistant to chemotherapy. Unlike other antimicrotubule drugs, such as* Vinca* alkaloids, which induce the disassembly of microtubules, taxanes promote the polymerization of tubulin. The microtubules formed in the presence of taxanes are extraordinarily stable and dysfunctional, causing cell death by disrupting the normal microtubule dynamics required for cell division and vital interphase processes. Therefore, taxanes are considered potent therapeutic agents against many cancers. They also enhance the cytotoxic effects of ionizing radiation in vitro, possibly by inducing cell cycle arrest in the G2/M phase, the most radiosensitive phase of the cell cycle [23, 24]. The results of some studies on esophageal cancer have suggested that the addition of taxanes to the standard preoperative regimen results in a significant improvement in the pCR rate [23]. Others have also reported that adding taxanes to standard regimens may benefit patients with esophageal cancer by improving survival and decreasing recurrence [4, 11, 25]. In contrast, other studies have reported that the addition of taxanes to the combined-modality regimen results in increased toxicity and fails to influence the overall survival, median survival time, or even the pCR rate [4, 26, 27]. Table 3 shows that the chemotherapeutic regimen is the factor most associated with overall survival. Other factors, including age, tumor location, post-CCRT pathologic T stage, CCRT response (pCR or MRD), and CCRT complications, do not significantly influence overall survival. The 12 cases of postoperative mortality may be indicative of the significant impact of postoperative complications on overall survival. Figure 1 shows that patients receiving taxane-incorporated CCRT had significantly better overall survival than those who did not (PF group).

Generally, the most important contributor to morbidity and mortality after esophagectomy is the development of complications, and these complications continue to be appreciably higher than other similarly complex operations, such as pancreatectomy, gastrectomy, and hepatectomy. Interestingly, our finding showed that patients who received taxane-incorporated CCRT have fewer postoperation complications than those who received PF regimen which may be due to promotion of tumor cells death to decrease complex operation level in our minimally invasive surgery (Table 2). Additionally, among the patients aged <60 (y/o), 71% (47/66) received the taxane-incorporated CCRT and 29% (19/66) received PF regimen (*P* = 0.001, Table 2). Although the patients aged <60 (y/o) were not statistically correlated with better outcome than the patients aged 60 (y/o) or more, the trend of clinical outcome has higher proportion of nonprogression in the patients aged <60 (y/o) than in the patients aged 60 (y/o) or more (42.4% versus 32.3%, Table 1). Therefore, the taxane-incorporated CCRT is the beneficial regimen in clinical outcome of esophageal squamous cell carcinoma patients potentially due to the decrease of postoperation complication (Table 2). However, only 11 taxane-incorporated CCRT patients initially received induction chemotherapy, which included modified TPFL (Taxotere, cisplatin, 5-FU, and leucovorin) or TP-HDFL (Taxotere, cisplatin, high-dose 5-FU, and leucovorin) according to other physicians, which is difficult for statistics of clinical outcome. One of the concerns regarding taxane-based CCRT is its moderate-to-severe toxicity, which has been previously reported. Neutropenia is known to be the principal toxic effect; however, several other effects were observed, including hematologic toxicity, hypersensitivity reaction, peripheral neurotoxicity, cardiac arrhythmia, gastrointestinal upset, and mucositis [3, 11, 24]. However, it remains unclear whether the frequency or severity of adverse events is higher with taxane-based chemotherapy than with PF. In our study, the CCRT complication rate was not significantly different between the treatment groups (Table 2), and, in our experience, the toxicity is manageable [3, 11]. However, a trend toward the use of taxanes (taxane-incorporated CCRT) in younger patients was noted in our study (Table 2), which may have had an influence on our analysis.

The limitations of this study are that the study is retrospective and is a single institutional study and data were missing in some of the studied cases. Another shortcoming is the lack of randomization. Specifically, the choices of the chemotherapeutic regimen and surgery were not random but depended on the physician's discretion. Although there was no statistically significant difference between the groups except for age (Table 2), selection bias can still be expected.

In conclusion, incorporation of taxane into cisplatin-based CCRT is potentially associated with better survival outcome in patients with pMR to neoadjuvant CCRT after esophagectomy. A lower rate of disease progression and comparable complication susceptibility were noted in this group of patients. However, these results need to be further examined in the future.

## Supplementary Material

Systemic recurrence (metastasis) sites were shown in Supplementary Table 1.

## Figures and Tables

**Table 1:** Characteristics of 117 patients stratified by disease progression.

Subgroup	Total *N* = 117	Disease progression (percentage) *n* = 72	Nonprogression (percentage) *n* = 45	*P* value
Age				
<60 y	66	38 (57.6)	28 (42.4)	0.316
≥60 y	51	34 (66.7)	17 (32.3)	
Gender				
Male	107	67 (62.6)	40 (37.4)	0.505
Female	10	5 (50.0)	5 (50.05)	
T staging^*∗*^				
0, 1, 2	107	64 (59.8)	43 (40.2)	0.313
3, 4	10	8 (80.0)	2 (20.02)	
CCRT response^*∗∗*^				
pCR	67	40 (59.7)	27 (40.3)	0.636
MRD	50	32 (64.0)	18 (36.0)	
CCRT regimen				
PF	50	39 (78.0)	11 (22.0)	0.002
Taxane-incorporated CCRT^*∗∗∗*^	67	33 (49.3)	34 (50.7)	

*P* value from *χ* ^2^ test or Fisher's exact test where the value is <0.05.

^*∗*^Post-CCRT pathologic T staging.

^*∗∗*^pCR: pathologic complete response; MRD: microscopic residual disease.

^*∗∗∗*^Taxane-incorporated CCRT: modified TPFL or TP-HDFL and then neoadjuvant CCRT (TP regimen), or the standard neoadjuvant CCRT with TP regimen.

**Table 2:** Characteristics of 117 patients stratified by chemotherapeutic regimen.

	Total *N* = 117	PF^*∗*^ (percentage) *n* = 50	Taxane-incorporated CCRT^*∗∗*^ (percentage) *n* = 67	*P* value
Age				
<60 y	66	19 (38.0)	47 (70.1)	0.001
≥60 y	51	31 (62.0)	20 (29.9)	
Gender				
Male	107	46 (92.0)	67 (91.0)	0.565
Female	10	4 (8.0)	6 (9.0)	
Site				
Upper thoracic	16	6 (12.0)	10 (14.9)	0.171
Middle thoracic	47	25 (50.0)	22 (32.8)	
Lower thoracic	54	19 (38.0)	35 (52.2)	
Op. complication				
No	61	21 (42.0)	40 (59.7)	0.058
Yes	56	29 (58.0)	27 (40.3)	
CCRT complication				
No	77	33 (66.0)	44 (65.78)	0.970
Yes	40	17 (34.0)	23 (34.3)	
Progression^*∗∗∗*^				
Nonprogression	45	11 (22.0)	34 (50.7)	0.002
Disease progression	72	39 (78.0)	33 (49.3)	

*P* value from *χ* ^2^ test or Fisher's exact test where the value is <0.05.

^*∗*^PF: cisplatin and 5-fluorouracil.

^*∗∗*^Taxane-incorporated CCRT: modified TPFL or TP-HDFL and then neoadjuvant CCRT (TP regimen), or the standard neoadjuvant CCRT with TP regimen.

^*∗∗∗*^The patients with postoperative mortality or cancer recurrence.

**Table 3:** Risk factors for overall survival and progression-free survival.

Variables	Overall survival				Progression-free survival			
	Crude HRs (95% CI)	*P* value	Adjusted HRs (95% CI)	*P* value	Crude HRs (95% CI)	*P* value	Adjusted HRs (95% CI)	*P* value
All (*N* = 119)								
Age								
<60	1		1		1		1	
≥60	1.11 (0.66–1.85)	0.696	0.78 (0.44–1.40)	0.411	1.11 (0.69–1.76)	0.672	0.86 (0.50–1.45)	0.564
T stage^*∗*^								
0, 1, 2	1		1		1		1	
3, 4	1.51 (0.65–3.51)	0.343	1.39 (0.56–3.43)	0.477	1.90 (0.90–3.99)	0.091	1.65 (0.74–3.67)	0.222
Tumor location								
Upper	1		1		1		1	
Middle	0.84 (0.39–1.82)	0.658	0.68 (0.31–1.52)	0.352	0.95 (0.46–1.95)	0.877	0.80 (0.38–1.71)	0.560
Lower	0.83 (0.39–1.77)	0.620	0.79 (0.36–1.73)	0.550	1.07 (0.53–2.17)	0.855	0.99 (0.48–2.06)	0.980
Op. complication								
No	1		1		1		1	
Yes	1.92 (1.14–3.23)	0.014	1.90 (1.09–3.32)	0.024	1.65 (1.04–2.63)	0.034	1.61 (0.98–2.65)	0.066
CCRT response^*∗∗*^								
pCR	1		1		1		1	
MRD	1.25 (0.74–2.10)	0.399	0.97 (0.53–1.78)	0.919	1.31 (0.82–2.09)	0.264	1.07 (0.62–1.84)	0.819
CCRT complication								
No	1		1		1		1	
Yes	0.76 (0.44–1.33)	0.335	0.80 (0.45–1.42)	0.444	0.88 (0.54–1.44)	0.610	0.97 (0.58–1.62)	0.905
CCRT regimen								
Taxane-incorporated CCRT^*∗∗∗*^	1		1		1		1	
PF	1.97 (1.16–3.35)	0.013	2.00 (1.13–3.55)	0.017	1.51 (0.94–2.42)	0.086	1.52 (0.91–2.53)	0.112

^*∗*^Post-CCRT pathologic T staging.

^*∗∗*^pCR: pathologic complete response; MRD: microscopic residual disease.

^*∗∗∗*^Taxane-incorporated CCRT: modified TPFL or TP-HDFL and then neoadjuvant CCRT (TP regimen), or the standard neoadjuvant CCRT with TP regimen.
