# Supplementary material for: Predictors of Survival in Esophageal Squamous Cell Carcinoma with Pathologic Major Response after Neoadjuvant Chemoradiation Therapy and Surgery: The Impact of Chemotherapy Protocols
Source: Biomed Res Int. 2016 Oct 4;2016:6423297. doi: 10.1155/2016/6423297 (PMC5061941; doi:10.1155/2016/6423297)
Supplement: Supplementary file 1 — Systemic recurrence (metastasis) sites were shown in Supplementary Table 1. [file 6423297.f1.docx]

| Supplementary table 1. Characteristics of 72 patients with disease progression after CCRT regimen. | | | | |
| --- | --- | --- | --- | --- |
|  |  | Disease progression  (Percentage)  n = 72 | |  |
| Subgroup | Total  N = 72 | Local recurrence  (N=24) | Systemic recurrence (metastasis)  (N=48) | *P*-value |
| Age |  |  |  |  |
| <60 y | 38 | 11 | 27 | 0.404 |
| ≥60 y | 34 | 13 | 21 |  |
| Gender |  |  |  |  |
| Male | 67 | 23 | 44 | 0.512 |
| Female | 5 | 1 | 4 |  |
| T staging* |  |  |  |  |
| 0, 1, 2 | 64 | 21 | 43 | 0.791 |
| 3, 4 | 8 | 3 | 5 |  |
| CCRT response** |  |  |  |  |
| pCR | 40 | 14 | 26 | 0.737 |
| MRD | 32 | 10 | 22 |  |
| Site |  |  |  |  |
| Upper thoracic | 10 | 4 | 6 | 0.776 |
| Middle thoracic | 28 | 10 | 18 |  |
| Lower thoracic | 34 | 10 | 24 |  |
| Op. complication |  |  |  |  |
| No | 34 | 12 | 22 | 0.738 |
| Yes | 38 | 12 | 26 |  |
| CCRT regimen |  |  |  |  |
| PF | 39 | 13 | 26 | 1.000 |
| Taxane-incorporated CCRT*** | 33 | 11 | 22 |  |
| Systemic recurrence (metastasis) sites |  |  |  |  |
| Lung |  |  | 8 |  |
| Brain |  |  | 4 |  |
| Bone |  |  | 9 |  |
| Liver |  |  | 9 |  |
| Others**** |  |  | 18 |  |

*P*-value from χ^2^ test or Fisher’s exact test where the value is <0.05

* Post-CCRT pathologic T staging.

** pCR: pathologic complete response, MRD: microscopic residual disease.

*** Taxane-incorporated CCRT: modified TPFL or TP-HDFL and then neoadjuvant

CCRT (TP regimen), or the standard neoadjuvant CCRT with TP regimen.

****Others: metastasis sites included scalp, retroperitoneum, gastric tube or multiple metastasis at the same time of daignosis.
